# The association between BMI and health-related physical fitness among Chinese college students: a cross-sectional study

**DOI:** 10.1186/s12889-020-08517-8

**Published:** 2020-04-05

**Authors:** Xiaobin Chen, Jie Cui, Yuyuan Zhang, Wenjia Peng

**Affiliations:** 1 Department of Sports and Art, Bengbu Medical College, Bengbu, Anhui China; 2 Department of Epidemiology and Health Statistics, School of Public Health, Bengbu Medical College, Bengbu, Anhui China

**Keywords:** College students, Body mass index, Physical fitness, Cross-sectional study

## Abstract

**Background:**

Existing studies reporting on the levels of physical fitness among college students used relatively few fitness tests as a reflection of physical fitness, which could not comprehensively evaluate the levels of physical fitness. Thus, the current study aimed to investigate the cross-sectional relationship between body mass index (BMI) and a physical fitness index (PFI) based on six indicators of fitness in Chinese college students.

**Method:**

Anthropometric measurements and six measures of physical fitness (Vital capacity, 50-m sprint, sit and reach, standing long jump, 800/1000-m run, pull-up/bent-leg sit-up) were measured. BMI was calculated to classify individuals into underweight, normal weight, overweight, and obesity groups. Z-scores based on sex-specific mean and standard deviation were calculated, and the sum of z-scores for the six fitness tests was used as a PFI. Three models (a linear regression model, polynomial regression model with a second-order BMI term and a restricted cubic spline regression model) were fitted to discuss the potential relation between BMI and PFI. We compared the models using Akaike Information Criterion (AIC) and R square.

**Results:**

Totally, 8548 freshmen from the years 2014 to 2016 in a medical college completed the physical fitness tests. There was a decreasing trend of physical fitness index from the years 2014 to 2016 (*P* for trend < 0.01). More male than female students were overweight or obese (23.5% vs. 11.9%), but more female than male students were normal weight (74.7% vs. 64.8%). A restricted cubic spline regression model was superior to linear and polynomial regression model with lower AIC and higher R square.

**Conclusions:**

The relationships between BMI and PFI in college students were non-linear. Underweight, overweight and obese students had poorer performance in physical fitness index than normal weight students. Future prospective, longitudinal cohort studies to identify the causal relations and potential mechanism in a good manner are required.

## Background

College is a transitional period from adolescence to adulthood, and is also a crucial period for the development of healthy lifestyles and the formation of healthy behaviors [1]. In recent years, there was a significant decline in physical activity among college students [2]. As reported by Hallal et al. [2], 31.1% of adults aged 15 years or older are not physically active all over the world. One study performed by Wang et al. [3] in Chinese adolescents found that the prevalence of physical activity time less than 1 hour per day was high in students aged 9–22 years. The highest prevalence of physical activity time less than 1 hour per day was 82.5% for 18 years old male students and 89.8% for 21 years old females, respectively. Maintaining physical activity has an important public health implication for the prevention of chronic diseases [4]. Physical activity has been found to be negatively associated with multiple diseases, such as cancer [5], obesity [6], diabetes [7], coronary artery disease [8] and depression [9] etc. The decrease in physical activity could lead to decreased physical fitness. Health-related physical fitness is also influenced by many other factors, such as total body fat and socioeconomic status. There are a few studies that discussed the association between body mass index (BMI) and several components of physical fitness in children [10] and in adolescents [11]. Studies on college students are relatively scarce in China. Existing studies reporting on the levels of physical fitness among college students used relatively few fitness tests as a reflection of physical fitness, which could not comprehensively evaluate the levels of physical fitness. Lu et al. [12] analyzed the 50-m test as an index of physical fitness. 50-m run test just reflected the speed and explosive strength of students. Hao et al. [13] used BMI, vital capacity index, sidestep test and standing long jump to investigate the gender differences in physical fitness. The purpose of this study is to analyze the levels of different physical fitness components in medical school freshmen, and to further evaluate the association between BMI and health-related physical fitness.

## Methods

The data were from a national survey on the physical fitness conducted among medical college freshmen from the years 2014 to 2016 in Anhui province, China. A sample aged 15–25 years was included to complete the physical fitness tests. All the participants provided written informed consent. For participants under the age of 16, both participants and their parents (or guardians) gave their informed consent. This study was approved by the Ethics Committee of Bengbu medical college (Ref No: 2018–050).

### Anthropometric measurements

Height and weight for each student were measured based on the protocol of National Health and Nutrition Examination Survey. The BMI was calculated according the following formula: BMI = Weight (kg) / height (m)^2^. BMI values were generally divided into four groups based on the criteria of World Health Organization (WHO): < 18.5 kg/m^2^, 18.5~23.9 kg/m^2^, 24~27.9 kg/m^2^, and ≥ 28 kg/m^2^, which represented low weight, normal weight, overweight and obesity, respectively.

### Physical fitness test

The physical fitness tests included vital capacity weight index, 50-m sprint, sit and reach, standing long jump, 800/1000-m run, pull-up, and bent-leg sit-up.

### Vital capacity weight index

Physiology was evaluated using the vital capacity index. Vital capacity was measured using the XF495-KDL model apparatus. Students were required to put their mouths into the blowpipe and stood before the apparatus to hold the handle properly. Then, students pressed the button, took a deep breath, and completely exhaled. The apparatus automatically calculated the maximal breathing capacity. Vital capacity weight index was equal to vital capacity divided by weight.

### 50-m sprint

50- m sprint was tested to evaluate the speed and explosive strength of students. When the investigator said, “go,” the subjects began the 50-m run. They finished the run as fast as they could. The time in minutes and seconds was recorded.

### Sit and reach

Sit and reach was conducted to assess low back flexibility. Each subject with barefoot sat on the test instrument and gradually reach forward as far as possible with knees extended. The test was recorded twice, and the better score was retained.

### Standing long jump

Standing long jump was conducted to assess lower-limb explosive strength. Each subject stood at the starting line and was asked to jump forward as far as they could. The distance was measured with meter from the starting line to the heel of the closest foot [14]. The test was recorded twice, and the better score was retained.

### 800/1000-m run

Each student stood at the starting line and was asked to complete the 800- or 1000- m as fast as they could. The time in minutes and seconds was recorded. All the female students did the 800-m run, and male students did the 1000-m run.

### Pull-up

Pull-up was used to evaluate the upper body muscular strength. The test was scored as the number of pull-ups. The subject jumped up and pulled the bars with both hands. After standing still, subjects pulled with both arms at the same time. All the male students did the test.

### Bent-leg sit-up

Each subject was instructed to lay on a mat with knees bent at 90 degrees, raise their upper body, and touch their knees with their elbows. The number of bent-leg sit-up completed in 1 minute was recorded. All the female students did the test.

Based on the results of all above tests, we also generated a gender-specific composite physical fitness score, defined as the sum of the standardized values (Z-score) of all six tests. Z scores of 50-m sprint and 800/1000-m run were reversed because of lower times reflected better performances.

### Data analysis

Data was collected and analyzed by R version 3.3.2 (University of Auckland, Oakland, New Zealand). The mean and standard deviation ($\overline{\mathrm{x}}\pm \mathrm{s}$) were adopted to describe the quantitative variables. Independent sample t-test or one-way analysis of variance (ANOVA) was conducted to compare mean difference among groups. When ANOVA was significant, further comprehensive comparison was done with SNK-q test. Frequencies and percentages were used to describe the qualitative variables. Difference among groups was compared using chi-square test.

The LMS method in the VGAM package was used to determine the trends of physical fitness index with study year. We also fitted three models to discuss the potential relation between BMI and physical fitness index: a) a linear regression model with BMI as the continuous predictor, b) a polynomial regression model with a second-order BMI term, and c) a restricted cubic spline regression model with three knots. We compared the models using Akaike Information Criterion (AIC) and R square. Statistical significance was set at a *P* value < 0.05.

## Results

### Basic characteristics of participants

Totally, 8548 freshmen from the years 2014 to 2016 in a medical college successfully completed the physical fitness test. The basic characteristics of participants grouped by year are listed in Table 1. The mean (SD) of physical fitness score from the years 2014 to 2016 were 0.315(2.070), 0.106(2.218) and − 0.374(2.261), respectively. The difference was significant (F = 74.936, *P* < 0.01), suggesting a decreasing trend of physical fitness index (*P* for trend <0.01).

**Table 1:** The basic characteristics of participants grouped by gender

Variables		Total		Male			Female		
	2014 (*n* = 2639)	2015 (*n* = 2872)	2016 (*n* = 3037)	2014 (*n* = 1105)	2015 (*n* = 1209)	2016 (*n* = 1316)	2014 (*n* = 1534)	2015 (*n* = 1663)	2016 (*n* = 1721)
Age (years) [n(%)]									
≤ 17	281 (10.65)	293 (10.20)	347 (11.43)	113 (10.23)	106 (8.77)	142 (10.79)	168 (10.95)	187 (11.24)	205 (11.91)
18	911 (34.52)	1065 (37.08)	1292 (42.54)	376 (34.03)	449 (37.14)	552 (41.95)	535 (34.88)	616 (37.04)	740 (43.00)
19	819 (31.03)	894 (31.13)	849 (27.96)	346 (31.31)	394 (32.59)	363 (27.58)	473 (30.83)	500 (30.07)	486 (28.24)
20	392 (14.85)	394 (13.72)	354 (11.66)	161 (14.57)	170 (14.06)	173 (13.15)	231 (15.06)	224 (13.47)	181 (10.52)
21	162 (6.14)	149 (5.19)	120 (3.95)	74 (6.70)	60 (4.96)	52 (3.95)	88 (5.74)	89 (5.35)	68 (3.95)
≥ 22	74 (2.80)	77 (2.68)	75 (2.47)	35 (3.17)	30 (2.48)	34 (2.58)	39 (2.54)	47 (2.83)	41 (2.38)
χ^2^(df;P)	59.266 (10;<0.001)			29.592 (10;0.001)			37.927 (10;<0.001)		
Post-hoc	P1 = 0.278; P2 < 0.001; P3 < 0.001			P1 = 0.204; P2 < 0.001; P3 = 0.020			P1 = 0.681; P2 < 0.001; P3 = 0.002		
Weight status [n(%)]									
Underweight	360 (13.64)	400 (13.93)	327 (10.77)	135 (12.22)	136 (11.25)	155 (11.78)	225 (14.67)	264 (15.87)	172 (9.99)
Normal	1915 (72.57)	2012 (70.06)	2097 (69.05)	740 (66.97)	802 (66.34)	809 (61.47)	1175 (76.60)	1210 (72.76)	1288 (74.84)
Overweight	283 (10.72)	346 (12.05)	453 (14.92)	173 (15.66)	195 (16.13)	241 (18.31)	110 (7.17)	151 (9.08)	212 (12.32)
Obesity	81 (3.07)	114 (3.97)	160 (5.27)	57 (5.16)	76 (6.29)	111 (8.43)	24 (1.56)	38 (2.29)	49 (2.85)
χ^2^(df;P)	54.420 (6;<0.001)			17.038 (6;0.009)			54.863 (6;< 0.001)		
Post-hoc	P1 = 0.087; P2 < 0.001; P3 < 0.001			P1 = 0.603; P2 = 0.002; P3 = 0.043			P1 = 0.045; P2 < 0.001; P3 < 0.001		
Physical fitness score Mean(SD)	0.315 (2.070)	0.106 (2.218)	− 0.374 (2.261)	0.29 (2.11)	0.07 (2.28)	−0.31 (2.34)	0.34 (2.04)	0.13 (2.17)	−0.42 (2.19)
F (df1;df2;P)	74.936 (2;8545;< 0.001)			21.955 (2;3627;< 0.001)			55.502 (2;4915;< 0.001)		
Post-hoc	P1 < 0.001; P2 < 0.001; P3 < 0.001			P1 = 0.024; P2 < 0.001; P3 < 0.001			P1 = 0.006; P2 < 0.001; P3 < 0.001		

Note: df represents degree of freedom; df1 and df2 represent num df and denom df of F test, respectively. P1 represents the *P* value for comparison between the year 2014 and 2015; P2 represents the *P* value for comparison between the year 2014 and 2016; P3 represents the *P* value for comparison between the year 2015 and 2016

The prevalence of overweight and obesity in male students (23.5%) was significantly higher (χ^2^ = 74.936, *P* < 0.01) compared with female students (11.9%). While, the prevalence of normal weight in male students (64.8%) was significantly lower (χ^2^ = 98.476, *P* < 0.01) compared with female students (74.7%). In both genders, a trend towards increased in the prevalence of overweight and obesity was observed with the increase/rise in study year.

### Analyses with health-related physical fitness score by weight status groups

There was a noticeable decrease in physical fitness scores in male college students from the years 2014 to 2016 (*P* for trend < 0.01), with 0.287(2.107), 0.074(2.280), − 0.309(2.267), respectively. Similar result was also observed in female college students, with 0.336(2.043), 0.128(2.173), − 0.423(2.194) of physical fitness scores from the years 2014 to 2016, respectively (*P* for trend < 0.01).

Table 2 and Table 3 provide the mean value of health-related physical fitness according to weight status in male and female groups, respectively. Overweight and obese students presented a higher performance in vital capacity in both genders, but a worse performance in 50-m sprint, sit and reach, standing long jump, 800/1000-m run in males (*P* < 0.05). Underweight and normal weight students were associated with a higher performance in standing long jump, 1000-m run and bent-leg sit-up in female students (*P* < 0.05). In both genders, significant differences on all health-related fitness test items among weight status groups were found (*P* < 0.05). For males, students with normal weight generally achieved better performance on physical fitness index than those of other weight status (*P* < 0.05). Female students with a normal weight have significantly higher physical fitness index than those with overweight or obesity (*P* < 0.05). However, physical fitness index did not differ between females with underweight and normal weight.

**Table 2:** Difference of health-related physical fitness stratified by BMI category in male groups [mean (SD)]

Items	Low (1)	Normal (2)	Overweight (3)	Obesity (4)	F	*P*	Post Hoc Multiple Comparison					
							1–2	1–3	1–4	2–3	2–4	3–4
Vital capacity	−0.45 (0.87)	− 0.05 (0.97)	0.35 (1.00)	0.44 (1.04)	75.793	<0.001	< 0.001	< 0.001	< 0.001	< 0.001	< 0.001	0.205
Vital capacity weight index	0.53 (1.02)	0.12 (0.95)	−0.40 (0.81)	−1.04 (0.77)	197.865	<0.001	< 0.001	< 0.001	< 0.001	< 0.001	< 0.001	< 0.001
50-m sprint	0.05 (1.05)	− 0.10 (0.96)	0.11 (0.95)	0.61 (1.13)	41.678	< 0.001	0.004	0.336	<0.001	<0.001	<0.001	< 0.001
Sit and reach	−0.10 (1.05)	0.02 (0.99)	0.02 (0.99)	−0.10 (1.00)	2.684	0.045	0.022	0.062	0.971	0.944	0.066	0.111
Standing long jump	0.04 (0.96)	0.07 (1.01)	−0.15 (0.92)	−0.38 (1.00)	21.212	<0.001	0.512	<0.001	<0.001	<0.001	<0.001	0.002
1000-m run	0.06 (0.93)	−0.17 (0.89)	0.22 (1.03)	1.02 (1.33)	129.826	<0.001	<0.001	0.008	<0.001	<0.001	<0.001	<0.001
Pull-up	0.21 (1.01)	0.14 (1.01)	−0.39 (0.82)	−0.79 (0.55)	113.937	< 0.001	0.186	<0.001	<0.001	<0.001	<0.001	<0.001
physical fitness index	−0.41 (3.02)	0.46 (3.03)	−0.50 (2.99)	−2.46 (3.17)	79.398	<0.001	<0.001	0.631	<0.001	<0.001	<0.001	<0.001

**Table 3:** Difference of health-related physical fitness stratified by BMI category in female groups [mean (SD)]

Items	Low (1)	Normal (2)	Overweight (3)	Obesity (4)	F	*P*	Post Hoc Multiple Comparison					
							1–2	1–3	1–4	2–3	2–4	3–4
Vital capacity	−0.26 (0.95)	−0.02 (1.00)	0.34 (0.92)	0.59 (1.05)	47.429	<0.001	<0.001	<0.001	<0.001	<0.001	<0.001	<0.001
Vital capacity weight index	0.46 (1.09)	0.01 (0.98)	−0.48 (0.71)	−0.90 (0.71)	120.015	<0.001	<0.001	<0.001	<0.001	<0.001	<0.001	<0.001
50-m sprint	−0.12 (0.98)	−0.02 (0.99)	0.20 (1.02)	0.42 (1.07)	16.207	<0.001	0.017	<0.001	<0.001	<0.001	<0.001	0.033
Sit and reach	−0.10 (1.04)	0.02 (1.00)	−0.03 (0.90)	−0.05 (1.02)	2.999	0.029	0.004	0.259	0.669	0.280	0.425	0.819
Standing long jump	0.11 (1.01)	0.01 (0.98)	−0.17 (1.08)	−0.36 (1.06)	12.14	<0.001	0.029	<0.001	<0.001	<0.001	<0.001	0.083
800-m run	−0.05 (0.98)	−0.07 (0.98)	0.34 (0.95)	1.00 (1.09)	63.139	<0.001	0.769	<0.001	<0.001	<0.001	<0.001	<0.001
bent-leg sit-up	0.04 (0.98)	0.02 (1.00)	−0.13 (1.01)	− 0.38 (1.05)	9.167	<0.001	0.757	0.005	<0.001	0.001	<0.001	0.017
physical fitness index	−0.04 (2.83)	0.13 (2.94)	−0.53 (2.98)	−1.62 (3.18)	18.745	<0.001	0.173	0.006	<0.001	<0.001	<0.001	<0.001

### Regression analysis of physical fitness index with BMI

**Fig. 1:**
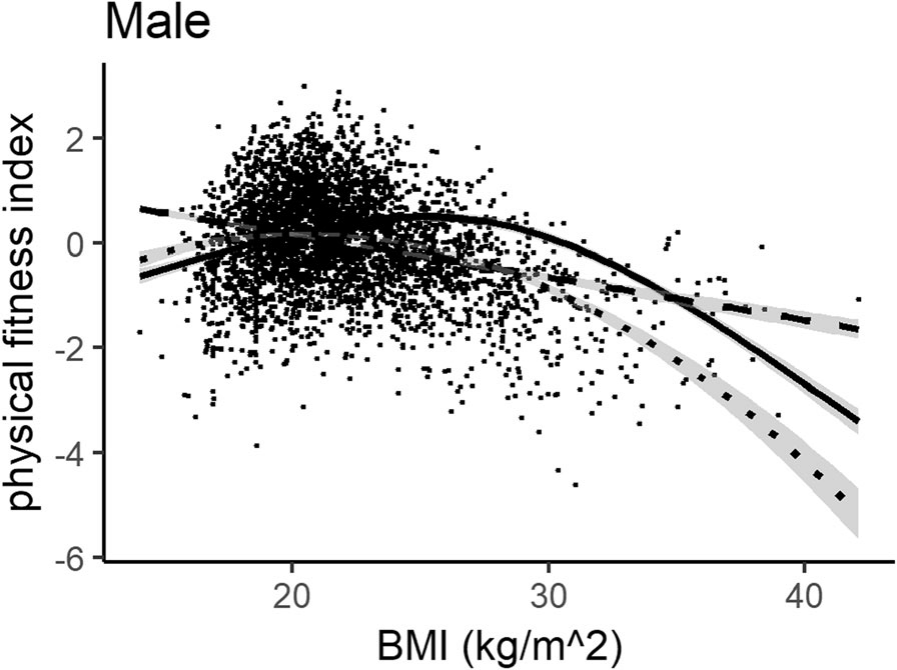
Linear regression, polynomial regression with second-order term, and restricted cubic splines (RCS) regression analyses with BMI and physical fitness in males. Dashed line: Linear regression (AIC: 9996.628, R-squared: 8.16%); Dotted line: polynomial regression (AIC: 9781.936, R-squared: 13.46%); Solid line: RCS regression (AIC: 9615.018, R-squared: 17.34%)

**Fig. 2:**
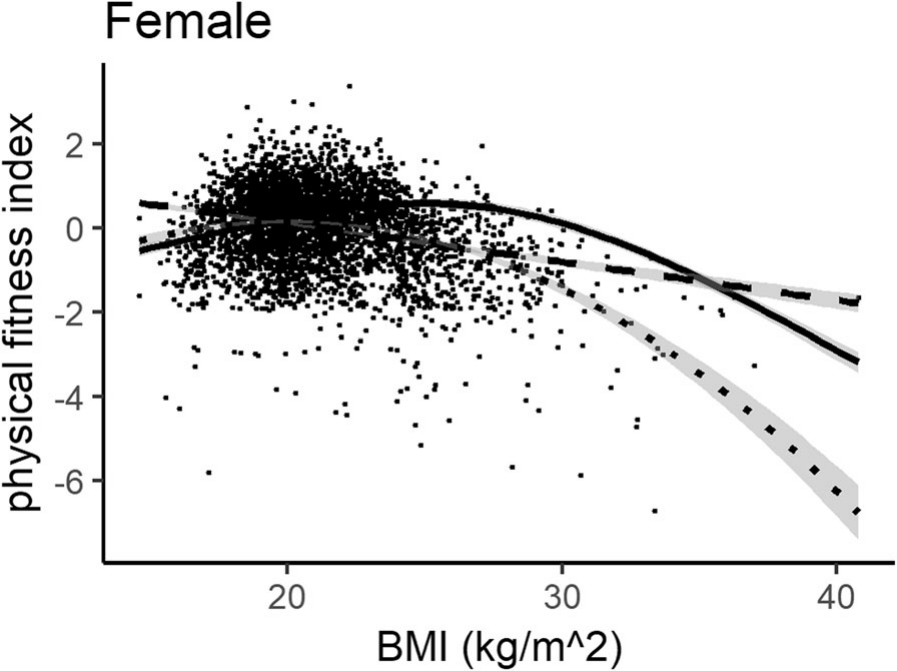
Linear regression, polynomial regression with second-order term, and restricted cubic splines (RCS) regression analyses with BMI and physical fitness in females. Dashed line: Linear regression (AIC: 10331.702, R-squared: 7.02%); Dotted line: polynomial regression (AIC: 10095.715, R-squared: 12.73%); Solid line: RCS regression (AIC: 9994.023, R-squared: 15.08%)

Figures 1 and 2 show the plots of the three regression models in the male and female groups, respectively. The AIC and R-squared values of the restricted cubic spline regression model were superior to the linear model and polynomial regression model with a second-order BMI term in both genders. Non-linearity was statistically significant (*P* < 0.01).

## Discussion

Physical fitness among college students is an important task of sport in schools, and is also one of the components of school physical education. Physical fitness aims to promote students to actively participate in physical exercise, and develop the habit of regular physical exercise, and improve self-health ability and physical health level.

Physical fitness has shown to be an important issue from a public health perspective [15]. The level of physical fitness was associated with health-related outcomes, including obesity, cardiovascular disease, skeletal health and mental health. With the development of society, the popularity and application of electronic products are becoming more and more widespread in the information era. There was a significant decline in physical activity among college students. A significant increasing trend of overweight and obesity was observed in the current cross-sectional study. The prevalence of overweight increased from 15.7 to 18.3% for males and from 7.2 to 12.3% for females during the years 2014 to 2016. For obesity in the same period, the prevalence of obesity increased from 5.2 to 6.7% for males and from 1.6 to 2.8% for females. Based on the data in the year 2014 from the national student physical fitness survey by the Ministry of Education of the People’s Republic of China once every 5 years, the obesity prevalence of students aged 19–22 years in both genders was slightly increased [16]. Although genetic factor plays an important role in obesity, the environmental and lifestyle factors such as physical activity and nutrition patterns are also considered to be of major importance [17]. Studies suggested that this increase could be attributed to rapid change of dietary and physical activity patterns [18].

The findings of the current study showed that overweight and obesity were more prevalent among male than female college students, while normal weight and underweight were more common among females, which was consistent with other studies [13, 19]. This gender difference is a common phenomenon in China. First, it could be explained by the differences in lifestyle. Males were more susceptible to unhealthy lifestyles, such as overeating and drinking. Second, females paid more attention to their body size and image, which made them tend to participate in activities to maintain their weight [20]. Besides, most females desired to be slim by dieting [21]. However, there was no significant gender difference in the United States [22]. On the contrary, overweight and obesity were found to increase only in females in South Africa [23].

Our findings suggested that overweight and obese students showed a higher performance in vital capacity compared with underweight and normal weight students. However, the abnormal weight status showed a bad performance of vital capacity weight index, which was in agreement with the study by Peng et al. [24].

Overweight and obese students achieved poorer performance in sit and reach, standing long jump, pull-up, sit-up, and endurance running compared with normal weight, which was consistent with the study by Mak et al. [25]. Obesity students might be less likely to take part in physical activity because of fear of poor performance and stigmatization [26]. However, Mak et al. [25] pointed out that the results should be interpreted with caution as overweight or obese subjects needed to use more energy to lift a greater body mass. What’s more, when adjusting for fat mass, the relations between overweight and obesity status and deficit in weight-bearing fitness tests were either attenuated or even reversed [15]. Underweight students were found to have higher performance in endurance running and bent-leg sit-up, but this was observed only in females in the present study.

Our results suggested that the relation between BMI and physical fitness was non-linear, which could be characterized by an inverted J-shape association. Polynomial regression and spline regression were clearly superior to the linear regression model, which was similar with Nikolakaros’s population-based study in Finnish healthy young men [27]. In our results, BMI explained around 17.34% of physical fitness variation in males by spline regression, 15.08% of physical fitness variation in females. Normal weight college students generally had better physical fitness than underweight, overweight and obese students, especially in males.

There are some limitations to the study that should be considered. Firstly, the sample could not truly represent the entire college students in China, as above 90% participants of the study were from Anhui province, which is located in east of China. However, the large sample size is one of the greatest strengths of this study. Secondly, the information on income level of their parents and residence (rural and urban, Southern and northern) were not obtained. As reported by Bohr et al. [28], girl students of lower socioeconomic status were associated with lower scores on the FITNESSGRAM assessments and less likely to achieve Healthy Fitness Zone status than those with higher socioeconomic status. Thirdly, the current study was a cross-sectional study. We could not establish a cause-and-effect relation but only identified association between BMI and physical fitness index.

## Conclusions

This study provided the evidence on the prevalence and trends of weight status and evaluated the relation between BMI and health-related physical fitness. Future prospective, longitudinal cohort studies to identify the causal relations and potential mechanism in a good manner are required.

## Data Availability

Available with the research team.

## Abbreviations

PFI: Physical fitness index
BMI: Body mass index
AIC: Akaike Information Criterion
RCS: Restricted cubic splines
